# IL-11 induces differentiation of myeloid-derived suppressor cells through activation of STAT3 signalling pathway

**DOI:** 10.1038/srep13650

**Published:** 2015-09-01

**Authors:** Kentaro Sumida, Yosuke Ohno, Junya Ohtake, Shun Kaneumi, Takuto Kishikawa, Norihiko Takahashi, Akinobu Taketomi, Hidemitsu Kitamura

**Affiliations:** 1 Division of Functional Immunology, Section of Disease Control, Institute for Genetic Medicine, Hokkaido University, Sapporo 06-0815, Japan; 2 Department of Gastroenterological Surgery I, Hokkaido University Graduate School of Medicine, Sapporo 060-8638, Japan

## Abstract

Myeloid-derived suppressor cells (MDSCs) are immune negative regulators in the tumour microenvironment. Interleukin (IL)-11, a member of IL-6 family cytokines, functions through the unique receptor IL-11 receptor α coupled with the common signal transducer gp130. IL-11-gp130 signalling causes activation of the JAK/STAT3 pathway. IL-11 is highly upregulated in many types of cancers and one of the most important cytokines during tumourigenesis and metastasis. However, the precise effect of IL-11 on differentiation into MDSCs is still unknown. Here, we found that CD11b^+^CD14^+^ monocytic MDSCs were generated from peripheral blood mononuclear cells (PBMCs) of healthy donors in the presence of IL-11. IL-11-conditioned PBMCs induced higher expression of immunosuppressive molecules such as arginase-1. A reduction of T-cell proliferation was observed when MDSCs generated in the presence of IL-11 were co-cultured with CD3/CD28-stimulated, autologous T cells of healthy donors. Culture of normal PBMCs with IL-11 led to STAT3 phosphorylation and differentiation into MDSCs via STAT3 activation. We confirmed expressions of both IL-11 and phosphorylated STAT3 in tumour tissues of colorectal cancer patients. These findings suggest that monocytic MDSCs may be induced by IL-11 in the tumour microenvironment. Thus, IL-11-mediated regulation in functional differentiation of MDSCs may serve as a possible target for cancer immunotherapy.

Activation of antitumor effectors is necessary for the suppression of tumour growth. However, it is difficult to induce tumour-specific T cell responses in tumour-bearing hosts, because they suffer from strong immunosuppressive tumour-escape mechanisms. Immunosuppressive factors, such as interleukin (IL)-6, IL-10 and Transforming growth factor (TGF)-β, impair the functions of T cells and dendritic cells in the tumour microenvironment^1^,^2^,^3^,^4^. Regulatory T cells (Tregs) are major immunosuppressors for T cell activation in tumour-bearing hosts. Indeed, several strategies attenuating Treg-mediated immune inhibition have been developed to enhance cytotoxic T lymphocyte (CTL)-mediated antitumor activity in tumour-bearing mice^5^,^6^,^7^.

Myeloid-derived suppressor cells (MDSCs) are important immune negative regulators in tumour hosts. In mice, these cells represent a heterogeneous population of CD11b^+^Gr-1^+^ cells that are classified into different subsets according to their phenotypic and morphological characteristics^8^,^9^,^10^,^11^,^12^,^13^. In human cancer patients, MDSCs are generally defined as monocytic (CD11b^+^, CD14^+^, HLA-DR^−/low^) or granulocytic (CD11b^+^, CD15^+^) myeloid cells with antitumor immunosuppressive function. These populations have been observed in the blood of patients with glioblastoma, lung cancer, breast cancer, colon cancer, or melanoma^14^,^15^,^16^,^17^. MDSCs inhibit efficient antitumor T cell responses via numerous factors and mechanisms, such as arginase-1, S100A8, S100A9, NADPH oxidase (NOX), reactive oxygen species (ROS), hydrogen peroxide (H_2_O_2_), and peroxynitrite (ONOO^−^)^18^,^19^,^20^,^21^,^22^,^23^. In addition, MDSCs have also been shown to suppress natural killer cell function^24^.

The IL-6 family is defined by the shared use of the gp130 receptor β-subunit. Included within this family is IL-6, recognized for its role in systemic inflammation and promoting platelet production^25^,^26^. More recently, IL-11 was reported to be involved in inflammation, angiogenesis and metastasis in tumour microenvironments^27^,^28^,^29^. IL-11 is produced by cancer-associated fibroblasts and myeloid cells in many types of tumour cells such as stomach, liver, pancreas, colon, ovary and breast cancer^28^,^29^,^30^,^31^,^32^,^33^,^34^,^35^,^36^,^37^. IL-11 as well as IL-6 activates STAT3, which is closely related to growth, differentiation and metastasis of cancer cells in the tumour microenvironment. However, no study has investigated the relationship between the antitumor immune response and IL-11-mediated activation of STAT3.

In this study, we report IL-11-STAT3-mediated regulation of functional differentiation of CD11b^+^CD14^+^ MDSCs. Indeed, a specific inhibitor of STAT3 significantly blocked the differentiation of induced MDSCs by IL-11. Moreover, PBMCs from healthy donors cultured with IL-11 acquired immunosuppressive activity. In this paper, we report a crucial role of IL-11 in the functional differentiation and immunosuppressive mechanisms of MDSCs, which may serve as a possible target for cancer immunotherapy.

## Results

### IL-11 generates CD11b^+^CD14^+^ monocytic immature cells from PBMCs

**Figure 1:**
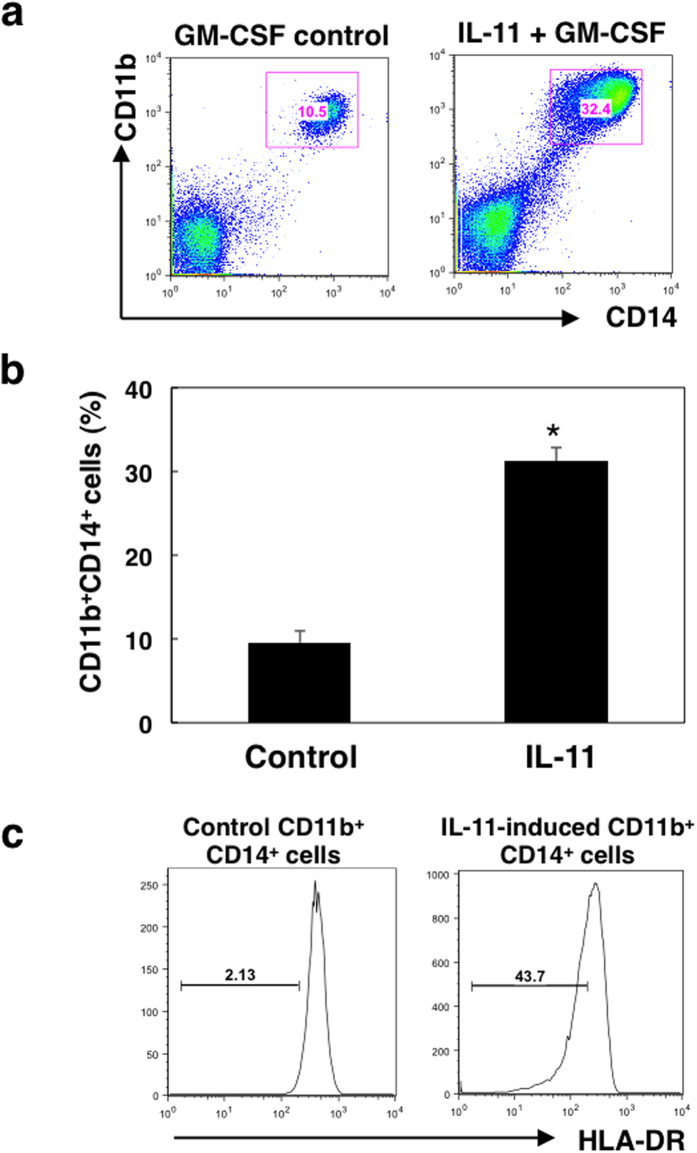
Effect of IL-11 on differentiation of PBMCs into CD11c^+^CD11b^+^ monocytic cells. PBMCs collected from blood of healthy donors were cultured with IL-11 (10 ng/ml) and GM-CSF (50 ng/ml) or GM-CSF alone for 7 days, and surface markers were analysed by flow cytometry. (**a**) Representative dot plots of CD11b^+^ and CD14^+^ cells. (**b**) Means and SDs for the data from three independent experiments are shown. *p < 0.05, compared with control, two-sided Student’s *t* test. (**c**) Surface expression levels of HLA-DR on CD11b^+^CD14^+^ cells were evaluated by flow cytometry. Bars represent HLA-DR negative and/or low populations. The representative of three independent experiments is shown.

To evaluate the effect of IL-11 on peripheral myeloid cells, we first investigated the differentiation of MDSCs from PBMCs cultured in the absence or presence of IL-11 for 7 days. As shown in Fig. 1a,b, percentages of CD11b^+^CD14^+^ monocytic populations were significantly increased by stimulation with IL-11. Furthermore, IL-11 increased CD11b^+^CD14^+^ monocytic populations in a dose-dependent manner (Supplementary Fig. S1), and CD11b^+^CD15^+^ granulocytic populations were not altered under these conditions (Supplementary Fig. S2). We also found that expression levels of IL-11R in the induced CD11b^+^CD14^+^ cells were enhanced in the presence of IL-11 (Supplementary Fig. S3). Furthermore, we confirmed that expression levels of HLA-DR were decreased in IL-11-conditioned CD11b^+^CD14^+^ cells (Fig. 1c). These data suggest that IL-11 may induce differentiation of PBMCs into monocytic MDSCs.

### IL-11-conditioned CD11b^+^CD14^+^ cells suppress T cell proliferation *in vitro*

**Figure 2:**
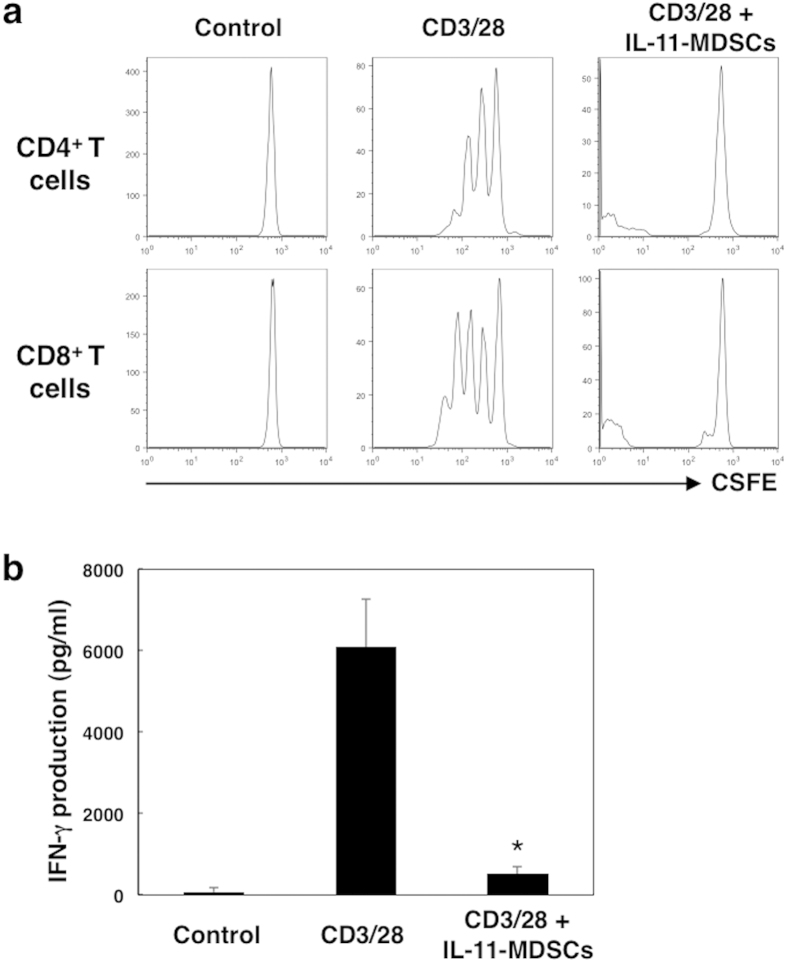
T cell activation in the presence of IL-11-induced CD11b^+^CD14^+^ cells. CD11b^+^CD14^+^ cells were generated from PBMCs in the presence or absence of IL-11 for 7 days. Isolated CD11b^+^CD14^+^ cells were co-cultured with CFSE-labelled, donor-matched autologous CD4^+^ and CD8^+^ T cells with or without T-cell receptor (TCR) stimulation by CD3/CD28 beads. (**a**) At 3 days after stimulation, T cell proliferation was determined by flow cytometric analysis of CFSE dilution. Representative histograms of the fluorescence intensity for CD4^+^ and CD8^+^ T cells without stimulation (control) or TCR-stimulated T cells cultured with or without CD11b^+^CD14^+^ cells. (**b**) IFN-γ production by T cells at 72 h after CD3/CD28 stimulation was measured by ELISA. The means and SDs of the data from three independent experiments are shown. *p < 0.05, compared with control, two-sided Student’s *t* test.

**Figure 3:**
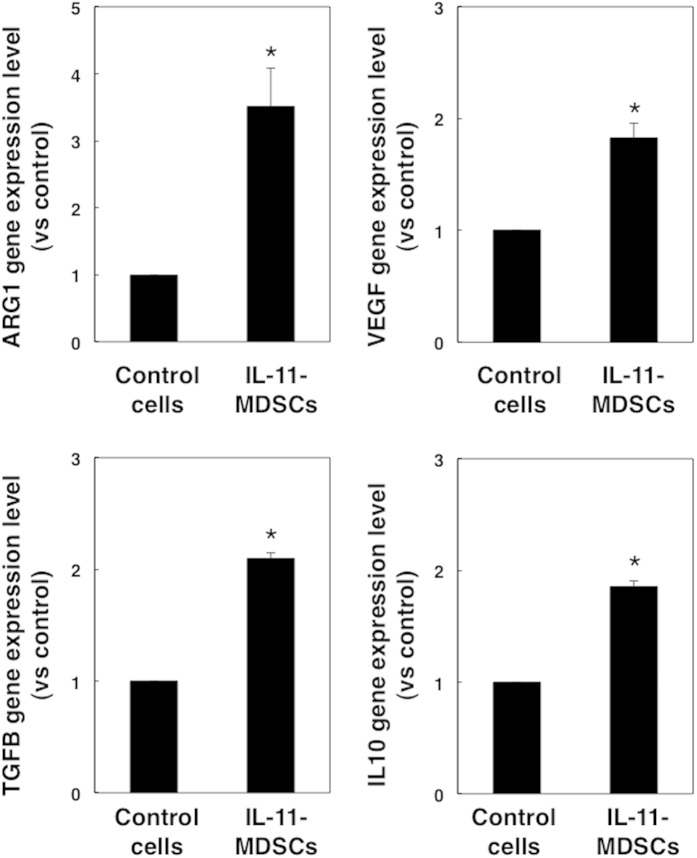
Gene expression levels of immunosuppressive molecules in IL-11-induced MDSCs. CD11b^+^ CD14^+^ cells were induced in the presence or absence of IL-11. Gene expression levels of ARG1, VEGF, TGF-β, and IL-10 in control CD11b^+^CD14^+^ cells and IL-11-induced CD11b^+^CD14^+^ MDSCs were evaluated by quantitative PCR. The means and SDs of the data from three independent experiments are shown. *p < 0.05, compared with control, two-sided Student’s *t* test.

Next we evaluated the immunosuppressive effects of CD11^+^CD14^+^ monocytic immature cells induced by stimulation with IL-11. The generated CD11^+^CD14^+^ cells were cultured with donor matched, autologous 5- or 6-Carboxyfluorescein diacetate succinimidyl ester (CFSE)-labelled CD3^+^ T cells in the presence of CD3/CD28 beads for 72 h. T cell proliferation was assayed by flow cytometry. A dramatic reduction of T cell proliferation was observed when CD11^+^CD14^+^ monocytic cells generated in the presence of IL-11 were co-cultured with CD3/CD28 stimulated, matched healthy donor CD4^+^ and CD8^+^ T cells (Fig. 2a). In addition, interferon (IFN)-γ production by T cells was significantly reduced by the addition of IL-11-induced MDSCs (Fig. 3b). These data suggest that IL-11-induced CD11^+^CD14^+^ monocytic immature cells may be involved in the induction of immunosuppression at tumour sites.

### IL-11-induced MDSCs upregulate arginase-1 gene expression

In addition to T cell inhibitory activity, we investigated the expression of immunosuppressive molecules in IL-11-induced MDSCs. We found that gene expression levels of arginase-1 were upregulated 3.5-fold in PBMCs cultured with IL-11, whereas VEGF (1.8-fold), TGF-β (2.1-fold), and IL-10 (1.9-fold) gene levels were only slightly enhanced (Fig. 3). From these data, we speculated that IL-11 induction at tumour sites is associated with enhanced expression of arginase-1, VEGF, TGF-β, and IL-10, which may be involved in the suppression of T cell responses in the tumour microenvironment.

### STAT3 activation is required for IL-11-induced generation of MDSCs

**Figure 4:**
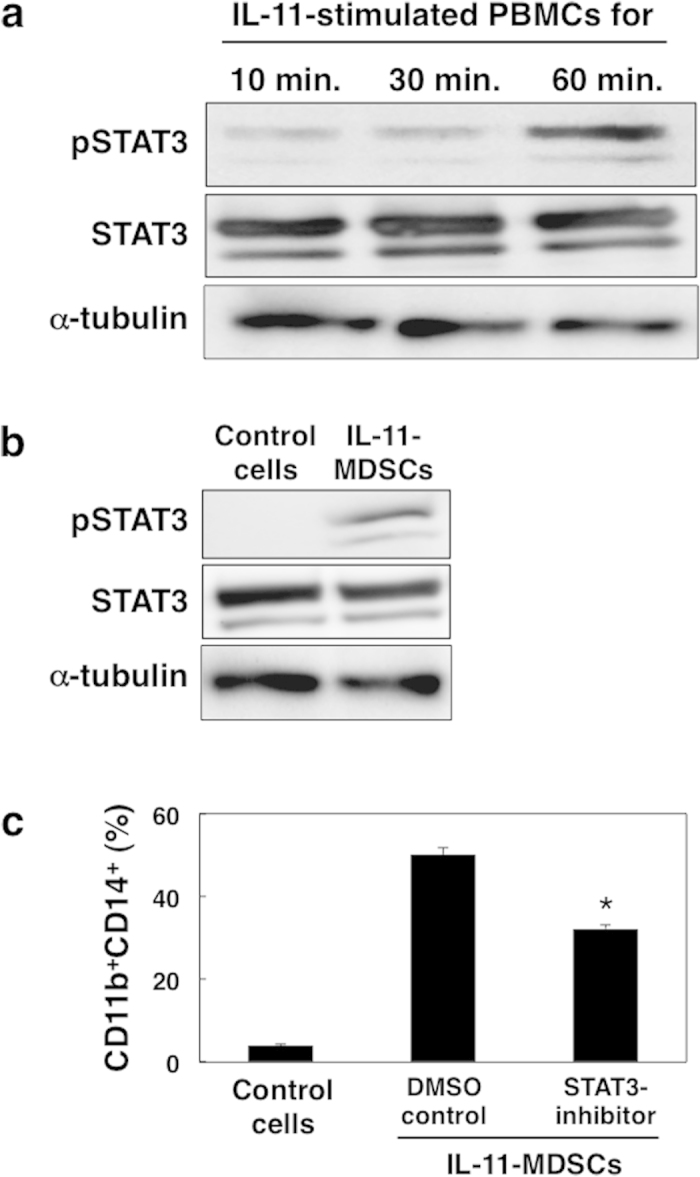
IL-11-dependent STAT3 activation in CD11b^+^CD14^+^ MDSCs. (**a**) PBMCs from blood of healthy donors were stimulated with IL-11 (10 ng/mL) for 10, 30, and 60 min. Total STAT3, phosphorylated STAT3 (pSTAT3), and alpha-tubulin proteins were evaluated by immunoblotting using specific antibodies. The representative data from three independent experiments are indicated. (**b**) PBMCs were cultured in the presence or absence of IL-11 for 7 days and CD11b^+^CD14^+^ cells were isolated by cell sorting. Total STAT3, pSTAT3, and alpha-tubulin were analysed by immunoblotting. The representative data from three independent experiments are indicated. c, PBMCs were cultured in the presence of STAT3 inhibitor (6-nitrobenzo[b]thiophene-1,1-dioxide, 10 μM) or DMSO with IL-11 and/or GM-CSF for 7 days. Percentages of the induced CD11b^+^CD14^+^ cells were determined by flow cytometry. The means and SDs of the data from three independent experiments are shown. *p < 0.05, compared with control, two-sided Student’s *t* test.

Activation of transcription factors such as STAT3 plays a role in regulating the differentiation of immature myeloid cells into MDSCs^26^. We next examined the role of the STAT3 signal transduction pathway in IL-11-mediated differentiation. We examined phosphorylation of STAT3 in MDSCs induced from PBMCs by IL-11, and found that IL-11 induced phosphorylation of STAT3 in PBMCs after 60 min of treatment (Fig. 4a). Moreover, we found that STAT3 was constantly activated in IL-11-induced MDSCs compared with control cells (Fig. 4b).

Next we investigated the requirement for STAT3 activation on differentiation of normal PBMCs into MDSCs by IL-11. We used the STAT3 inhibitor (6-nitrobenzo[b]thiophene-1,1-dioxide) and confirmed reduced phosphorylation of STAT3 (Supplementary Fig. S4). The percentage of MDSCs generated from PBMCs was significantly reduced in the presence of STAT3 inhibitor compared with dimethyl sulfoxide (DMSO) control (Fig. 4c). These data suggest that IL-11 functions in the differentiation of MDSCs via activation of the STAT3-signalling pathway.

### IL-11/STAT3 axis is involved in the tumour microenvironment in colorectal cancer patients

**Figure 5:**
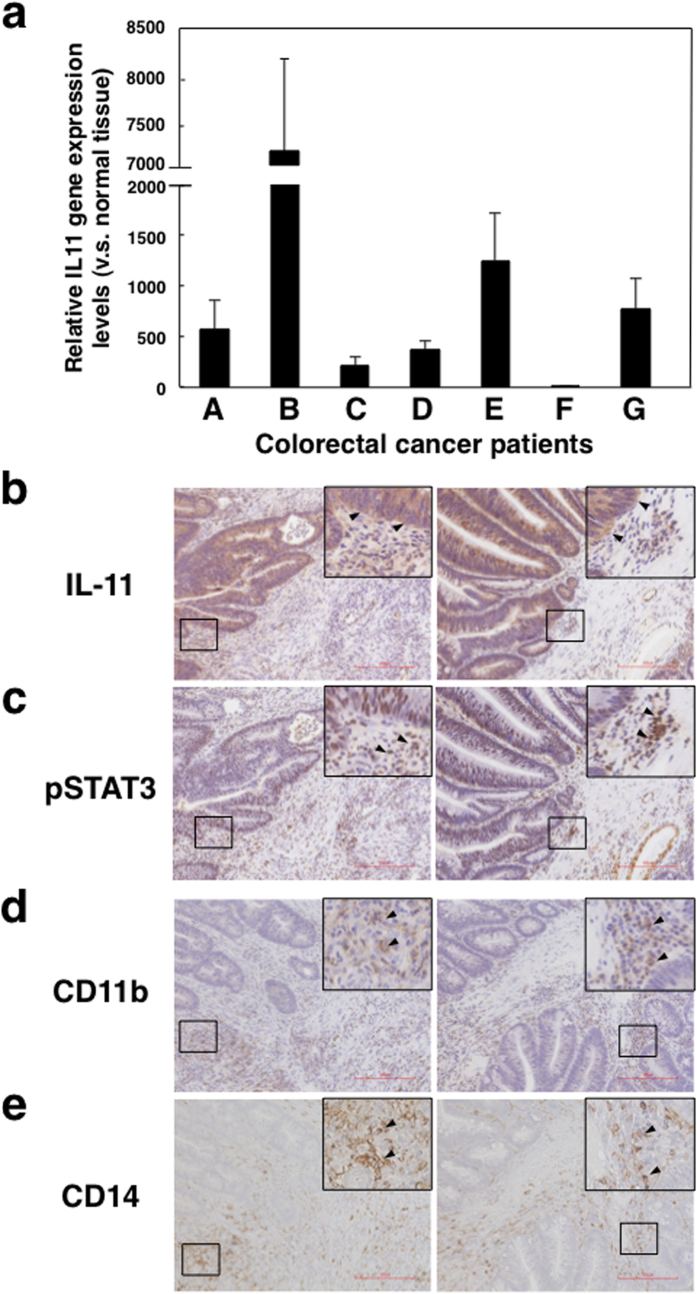
IL-11 and phosphorylated STAT3 expression in tumour microenvironments of colorectal cancer patients. (**a**) Normal and tumour tissues were collected from the specimens of seven colorectal cancer patients. Gene expression levels of IL-11 and GAPDH in normal and tumour tissues were determined by quantitative PCR. IL-11 gene expression in each sample was normalized to levels of GAPDH. Relative IL-11 gene expression levels of tumour tissues against normal tissues were calculated. The means and SDs of the data from three independent experiments are shown. *p < 0.05, compared with control, two-sided Student’s *t* test. IL-11 (**b**), pSTAT3 (**c**), CD11b (**d**), and CD14 (**e**) protein expressions in tumour tissues of colorectal cancer patients were detected by immunohistochemistry. Scale bar is 200 μm for all panels. Representative photos including magnified views and allows of patient B and E are shown.

Elevated STAT3 activation is associated with MDSCs and poor survival in cancer patients^36^. However, the role of IL-11 in MDSCs in tumour microenvironments is unknown. Here we investigated IL-11 gene expression in seven primary tumour tissues of patients with colon cancer. We found that expression of IL-11 gene was increased in tumour tissues compared with normal areas of tissues from the same patients (Fig. 5a). Furthermore, we confirmed that IL-11 protein was also expressed in tumour tissues of colorectal cancer patients (Fig. 5b).

Next we assessed phosphorylated STAT3 (pSTAT3) staining as a marker of activated STAT3 in the primary human colon cancer samples. We found strong pSTAT3 signals in the same region on adjacent sections of tumour tissues (Fig. 5c). Furthermore, we confirmed that CD11b^+^ and/or CD14^+^ cells infiltrated in the same region of tumour tissues of colorectal cancer patients (Fig. 5d,e). These observations suggest that IL-11-mediated STAT3 activation may be associated with differentiation of CD11b^+^CD14^+^ MDSCs in the tumour microenvironment of colorectal cancer patients.

## Discussion

Overcoming immunosuppressive tumour escape mechanisms is essential to induce tumour-specific CTLs to establish more efficient tumour immunotherapies. A recent report indicated that dietary iron enhanced the colonic IL-6/IL-11-STAT3 signalling pathway and promoted inflammation and subsequent tumour development in a mouse model of inflammation-associated colorectal tumourigenesis using DSS^38^. In addition, STAT3 activation directly promotes survival, proliferation, and differentiation of tumour cells and MDSCs. A previous study also demonstrated that IL-6 is a key cytokine that induces MDSCs under tumour-bearing conditions^36^. However, the precise mechanism underlying the effect of IL-11 on the generation of MDSCs was previously unknown. Here we demonstrated that IL-11 was produced as a soluble factor in the tumour microenvironment of patients with colon cancer (Fig. 5a,b) and promoted the differentiation of immune cells into functional MDSCs (Fig. 2). Although we have to investigate whether IL-11 from tumours directly affect the generation of MDSCs or not, we speculate that MDSCs were induced by IL-11 from the local tumour microenvironment, which was related with the immunosuppression at tumour sites. These data are consistent with recent reports demonstrating that exposure of MDSCs to either the tumour microenvironment or inflammatory site enhances suppressive functions^39^,^40^.

The STAT3 signalling pathway regulates various genes involved in the growth, proliferation, survival and differentiation of MDSCs as well as numerous types of cells^2^,^3^,^22^. In this study, we found that STAT3 was activated in IL-11-induced MDSCs (Fig. 3b). Moreover, we confirmed that the IL-11-mediated differentiation of MDSCs was blocked by the inhibition of STAT3 activation (Fig. 4c). The observed IL-11 expression and STAT3 activation in CD11b^+^ and/or CD14^+^ cell-infiltrating tumour sites of colorectal cancer patients (Fig. 5) strongly suggests that the induction of MDSCs may be mediated by IL-11-STAT3 signalling in the tumour microenvironment. We also evaluated MDSC induction in the presence of MEK inhibitor, U0126, which also blocked Ras/Erk and mTor signalling pathways, and found that generation of CD11b^+^CD14^+^ cells was reduced in the presence of the inhibitor (Supplementary Fig. S5). Therefore, we speculated that Ras/Erk and mTor pathways are partially involved in IL-11-induced MDSC generation. Our data may support a previous report^27^ indicating that IL-11 promotes tumorigenesis by induction of MDSCs through STAT3 activation.

In addition to the direct cytotoxic or cytostatic effects of conventional chemotherapeutic drugs and agents on target cancer cells, a recent paper demonstrated that these drugs could also promote the elimination or inactivation of suppressive Tregs or MDSCs, resulting in enhanced antitumor immunity^41^. Moreover, preclinical cancer mouse models indicate that blockade of IL-6- and IL-11-STAT3 signalling cascades is a promising strategy in development of cancer therapies^42^.

Our present data suggest that IL-11-induced MDSCs may play a role in the immunosuppression of tumour microenvironments. These findings suggest that IL-11-STAT3-mediated regulation of the functional differentiation of MDSCs may serve as a possible target for effective immunotherapy for cancers. Given the critical roles of MDSCs in immunosuppression, our elucidation of the effector functions in IL-11-induced MDSCs has implications in the rational design of strategies for enhancing antitumor immune responses and development of new efficient treatments for cancer patients.

## Methods

### Informed consent and approval

Research protocols involving healthy donors and colorectal cancer patients were approved by the Institutional Review Boards of Hokkaido University Graduate School of Medicine and the Institute for Genetic Medicine. Written informed consent was obtained from each patient and healthy donor. All protocols were approved by the Ethics Committee of Hokkaido University Graduate School of Medicine and Institute for Genetic Medicine in light of the Declaration of Helsinki. The methods were carried out in accordance with the approved guidelines.

### Preparation of CD11b^+^CD14^+^ cells from PBMCs

PBMCs were isolated from healthy donors by Ficoll-Paque (GE Healthcare, USA) and cultured in 10% fetal bovine serum and 100 mg/mL penicillin/streptomycin in RPMI 1640 (Wako, Japan). PBMCs were cultured with IL-11 (10 ng/ml) and/or GM-CSF (50 ng/ml) for 7 days, in the presence or absence of STAT3 inhibitor (6-nitrobenzo[b]thiophene-1,1-dioxide, 10 μM, Calbiochem). Cultured cells were stained with PE-conjugated anti-CD11b (Bear1, Beckman Coulter, Japan), fluorescein-isothiocyanate-conjugated anti-CD14 (M5E2, BD Biosciences, USA) and allophycocyanin (APC)-conjugated anti-CD15 (M2E2, BD Biosciences) monoclonal antibodies (mAbs). Dead cells were excluded by 7-AAD (Beckman Coulter) staining. The percentages of CD11b^+^CD14^+^ or CD11b + CD15^+^ cells were analysed by a FACSCanto II (BD Biosciences) and FlowJo software (Treestar). CD11b^+^CD14^+^ cells were isolated using a FACSAria (BD Biosciences). The purity of the isolated cells was consistently higher than 95%.

### T cell proliferation assay

CD3^+^ T cells were sorted from PBMCs with FACSAria. T cells were labelled with CFSE (Invitrogen) and cultured with CD3/CD28 beads (Invitrogen) for 3 days. Cells were collected, stained for CD4^+^ or CD8^+^ T cell markers and evaluated by flow cytometric analysis on a FACS Canto II. Cells were gated on CD4^+^ or CD8^+^ T cells and proliferation was determined on the basis of CFSE dilution.

### Enzyme-linked immunosorbent assay (ELISA)

IFN-γ levels in culture supernatants were measured by OptEIA™ human IFN-γ ELISA kits (BD Biosciences), according to the manufacturer’s instructions.

### Reverse-transcription polymerase chain reaction (RT-PCR)

Total RNA was extracted from cells using an Isogen RNA extraction kit (Qiagen, Germany). cDNA was prepared using Superscript III RT (Invitrogen). The indicated cDNAs were specifically amplified using a LightCycler system (Roche Applied Science) and the corresponding primer pairs and probes. The sequences were as follows: IL-11, (sense) 5′-ctgtggggacatgaactgtg-3′, (antisense) 5′-agggtctggggaaactcg-3′, and probe #49; Arg-1, (sense) 5′-tggcagaagtcaagaagaacg-3′, (antisense) 5′-atgcttccaattgccaaact-3′, and probe #64; VEGF, (sense) 5′-ccttgctgctctacctccac-3′, (antisense) 5′-ccacttcgtgatgattctgc-3′, and probe #29; TGF-β, (sense) 5′-actactacgccaaggaggtcac-3′, (antisense) 5′-tgcttgaacttgtcatagatttcg-3′, and probe #21; IL-10, (sense) 5′-gatgccttcagcagagtgaa-3′, (antisense) 5′-gcaacccaggtaacccttaaa-3′, and probe #67; and GAPDH, (sense) 5′-agccacatcgctcagacac-3′, (antisense) 5′-gcccaatacgaccaaatcc-3′, and probe #60. Samples were normalized to the housekeeping gene β-actin according to the ΔΔCt method: ΔCt = ΔCt_sample_ − ΔCt_reference_.

### Immunoblotting

The isolated cells were lysed in a buffer consisting of 20 mM HEPES, pH 7.5, 100 mM NaCl, 1.5 mM MgCl_2_, 1 mM EGTA, 10 mM Na_4_P_2_O_7_, 1% Nonidet P-40, 2 mM dithiothreitol, 1 mM vanadate, 1 mM phenylmethylsulfonyl fluoride, 2 mg/ml aprotinin, and 10% glycerol. The cell lysates were subjected to sodium dodecyl sulfate polyacrylamide gel electrophoresis and transferred to polyvinylidene difluoride membranes (Merck Millipore, Germany). Membranes were blocked with Blocking One (Nacalai Tesque, Japan) and probed with anti-STAT3 (79D7), anti-p-STAT3 (Tyr705) Abs (Cell Signaling Technology, USA), or anti-alpha-tubulin (T6199) Ab (Sigma-Aldrich). Membranes were washed and incubated with a secondary Ab conjugated with peroxidase. The protein levels were detected using an Image Quant LAS4000 mini (GE Healthcare) with ECL Plus (GE Healthcare).

### Immunohistochemistry

Cancer tissues were obtained from colorectal surgical specimens (n = 7). The tumors of all colorectal cancer patients were low grade (well or moderately differentiated). Colon specimens were fixed in formalin and embedded in paraffin. After deparaffinization, antigen retrieval was conducted at 95 °C for 20–30 min in EDTA buffer (pH 9.0). Endogenous peroxidase activity was blocked with 0.3% H_2_O_2_ at room temperature for 10 min. After protein blocking at room temperature for 10 min, slides were incubated with a polyclonal rabbit anti-human IL-11 (RPA057Hu01, Uscn Life Science, USA), a polyclonal rabbit anti-human phospho-STAT3 (Tyr705) antibody (9145, Cell Signaling Technology), a monoclonal mouse anit-CD14 antibody (MY4), or a rabbit monoclonal anti-CD11b antibody (EPR1344, Abcam, Cambridge, UK) overnight at 4 °C. Sections were then incubated at room temperature for 15 min with a horseradish peroxidase-labelled anti-rabbit Igs. Positive signals were amplified with the CSA II Biotin-free Tyramide Signal Amplification System (Dako, Japan) and visualized using 3-3′-diaminobezidine-4HCL (DAB). Hematoxylin-eosin staining was then performed according to standard procedures.

### Statistical analyses

Significant differences were determined using the two-sided Student’s *t* test. A value of *P* < 0.05 was considered significant.

## Additional Information

**How to cite this article**: Sumida, K. *et al.* IL-11 induces differentiation of myeloid-derived suppressor cells through activation of STAT3 signalling pathway. *Sci. Rep.* **5**, 13650; doi: 10.1038/srep13650 (2015).

## Supplementary Material

Supplementary Information
