# Supplementary material for: IL-11 induces differentiation of myeloid-derived suppressor cells through activation of STAT3 signalling pathway
Source: Sci Rep. 2015 Sep 1;5:13650. doi: 10.1038/srep13650 (PMC4642551; doi:10.1038/srep13650)
Supplement: Supplementary Information [file srep13650-s1.pdf]

# Supplementary Information

# IL-11 induces differentiation of myeloid-derived suppressor cells through activation of STAT3 signalling pathway

Kentaro Sumida, Yosuke Ohno, Junya Ohtake, Shun Kaneumi, Takuto Kishikawa, Norihiko Takahashi, Akinobu Taketomi, Hidemitsu Kitamura

Supplementary Figure S1

Supplementary Figure S2

Supplementary Figure S3

Supplementary Figure S4

Supplementary Figure S5

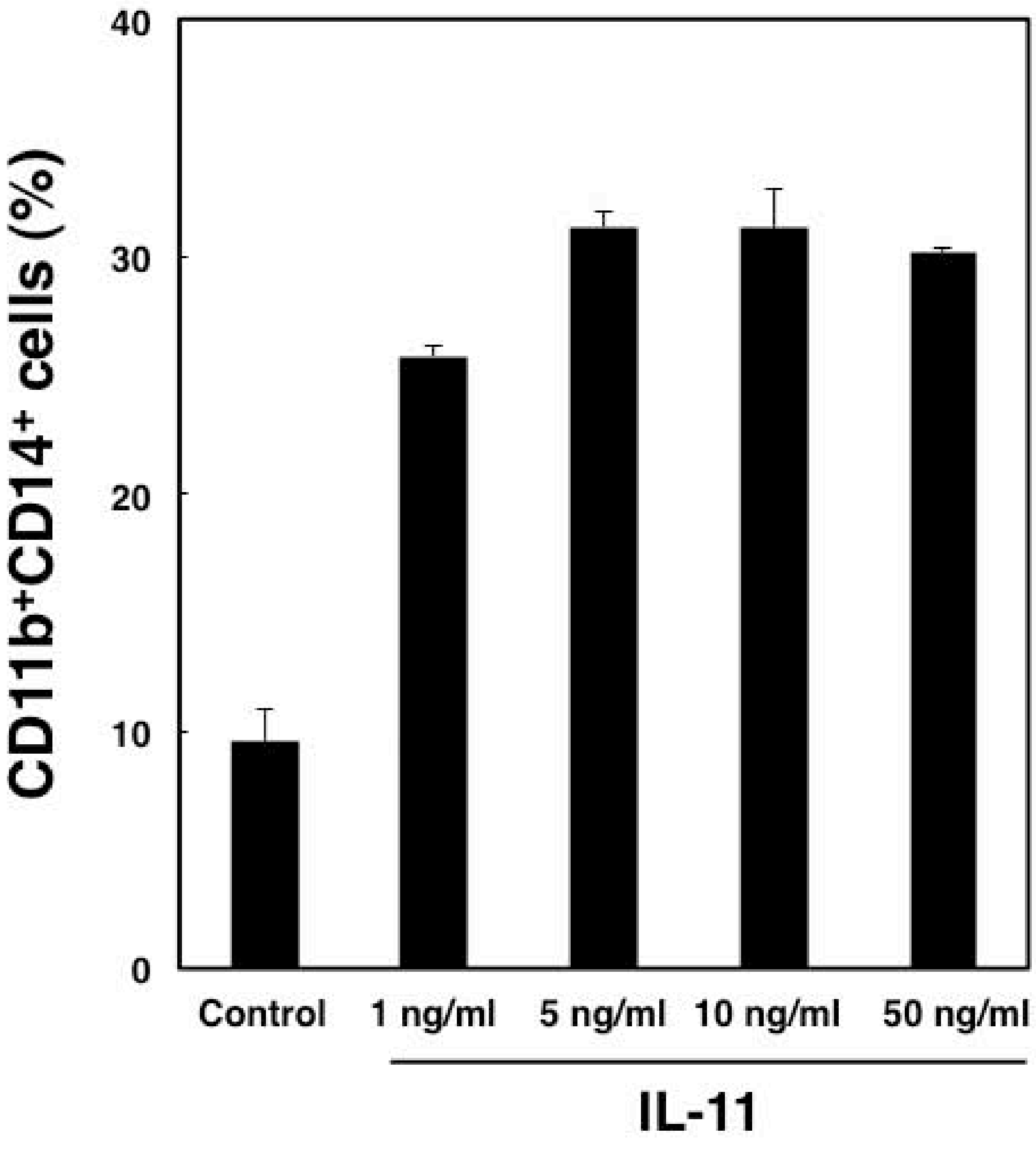


**Figure S1: Dose-dependent induction of $CD11b^{+}CD14^{+}$ cells in the presence of IL-11.**

PBMCs collected from blood of healthy donors were cultured with IL-11 (1, 5, 10, and 50 ng/ml) and GM-CSF (50 ng/ml) or GM-CSF alone for 7 days, and surface expression levels of CD11b and CD14 were analyzed by flow cytometry. Means and SDs for the representative data from three independent experiments are shown.

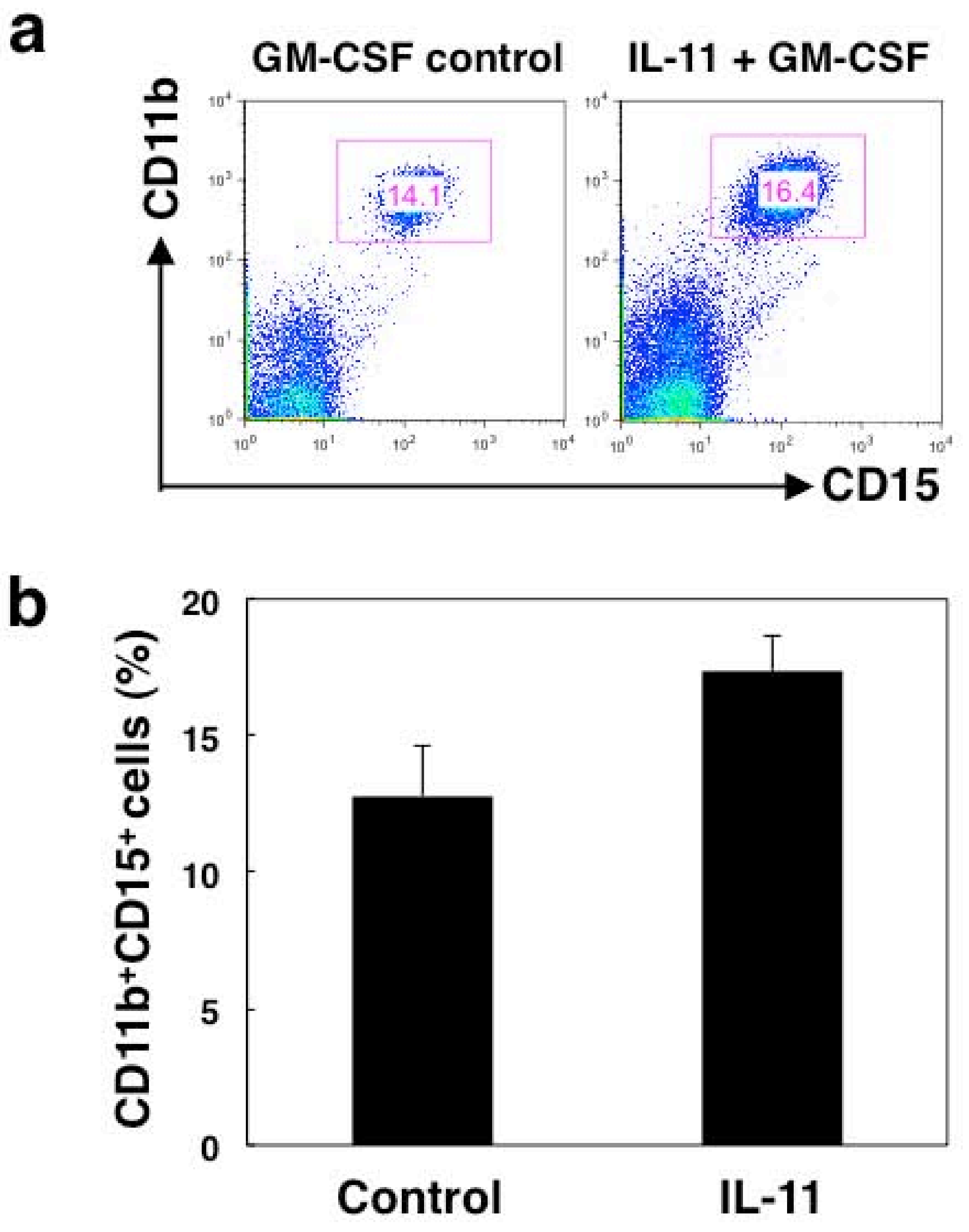


**Figure S2: CD11b⁺CD15⁺ cells were not altered in the presence of IL-11.** PBMCs collected from blood of healthy donors were cultured with IL-11 (10 ng/ml) and GM-CSF (50 ng/ml) or GM-CSF alone for 7 days, and surface expression levels of CD11b and CD15 were analyzed by flow cytometry. a, Representative dot plots of CD11b⁺ and CD15⁺ cells. b, Means and SDs for the representative data from three independent experiments are shown.

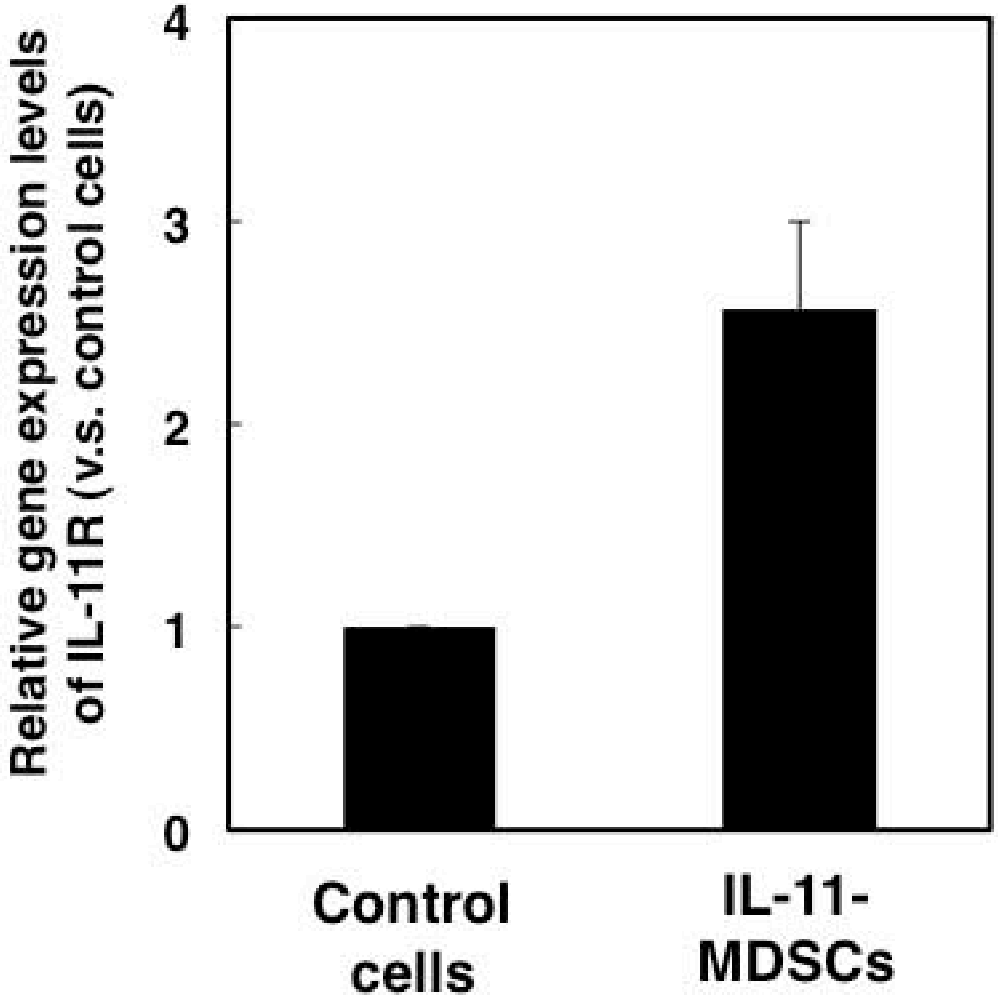


**Figure S3: IL-11R gene expression level was enhanced in IL-11-induced CD11b$^{+}$CD14$^{+}$ cells.**
CD11b$^{+}$CD14$^{+}$ cells were induced from PBMCs of healthy donors in the presence or absence of IL-11. Relative gene expression levels of IL-11R in control CD11b$^{+}$CD14$^{+}$ cells and IL-11-induced CD11b$^{+}$CD14$^{+}$ cells were evaluated by quantitative PCR. Means and SDs of the representative data are shown in the figure.

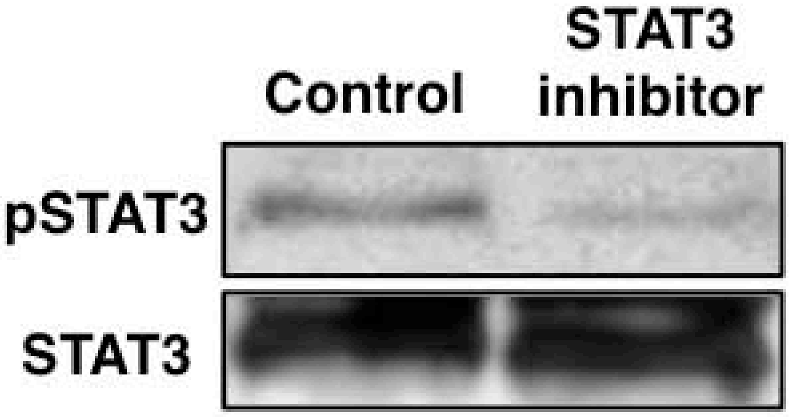


**Figure S4: STAT3 inhibitor suppresses phosphorylation of STAT3 in IL-11-induced CD11b$^+$CD14$^+$ cells.**

PBMCs were cultured in the presence of STAT3 inhibitor (6-Nitrobenzo[b] thiophene-1,1-dioxide, 10 $\mu$M, Calbiocham) or DMSO with IL-11 and GM-CSF for 7 days. CD11b$^+$CD14$^+$ cells were isolated by cell sorting. Total STAT3 and pSTAT3 were analyzed by immunoblotting. The representative data are indicated in the figure.

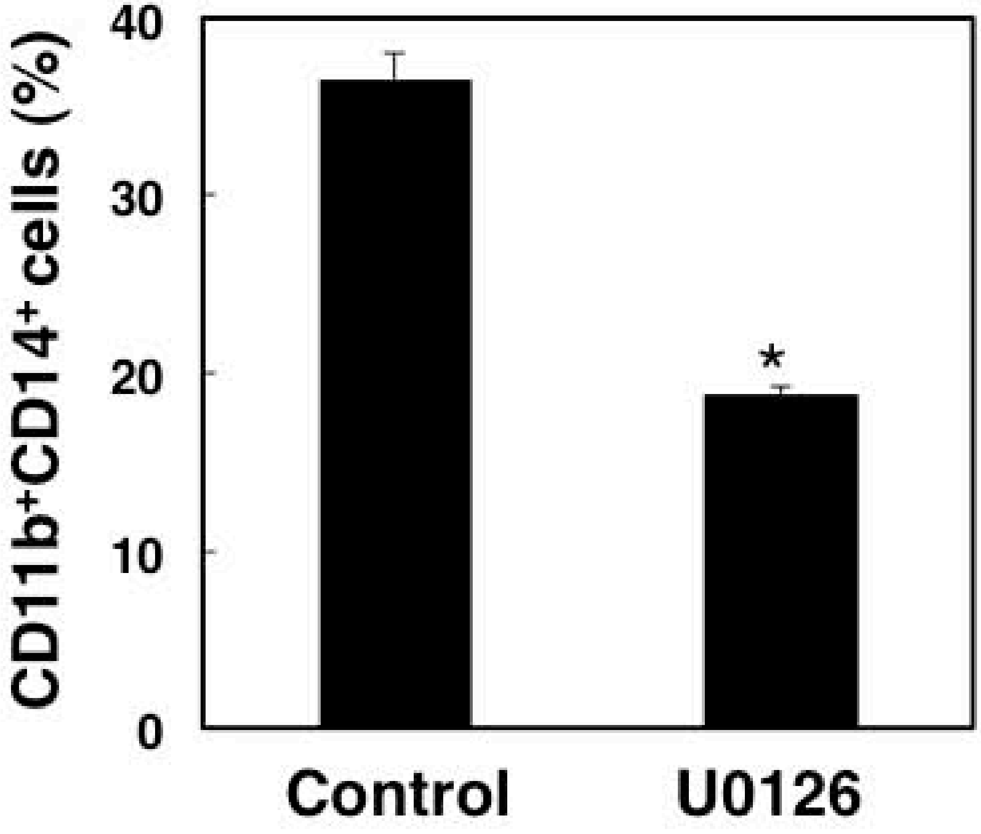


**Figure S5: Induction of $CD11b^+CD14^+$ cells by IL-11 in the presence of MEK inhibitor.**

PBMCs were cultured in the presence of MEK Inhibitor U0126 (10 μM) or DMSO with IL-11 and GM-CSF for 7 days. Percentages of the induced $CD11b^+CD14^+$ cells were determined by flow cytometry. Means and SDs of the representative data are shown in the figure.
